# A comprehensive Mendelian randomization study highlights the relationship between psychiatric disorders and non-tumor gastrointestinal diseases

**DOI:** 10.3389/fgene.2024.1392518

**Published:** 2024-05-09

**Authors:** Xiru Liang, Xindi Huang, Yutong Cheng, Ziwei Wang, Yahua Song, Qiuai Shu, Ning Xie

**Affiliations:** ^1^ Department of Gastroenterology, The Second Affiliated Hospital of Xi’an Jiaotong University, Xi’an, Shaanxi, China; ^2^ Shaanxi Key Laboratory of Gastrointestinal Motility Disorders, Xi’an Jiaotong University, Xi’an, Shaanxi, China; ^3^ The Key Laboratory of Biomedical Information Engineering of Ministry of Education, School of Life Science and Technology, Xi’an Jiaotong University, Xi’an, Shaanxi, China; ^4^ Bioinspired Engineering and Biomechanics Center (BEBC), Xi’an Jiaotong University, Xi’an, Shaanxi, China

**Keywords:** causality, GWAS, psychiatric disorders, non-tumor gastrointestinal diseases, Mendelian randomization

## Abstract

**Objective:**

Previous observational studies revealed the potential correlation between psychiatric disorders (PDs) and non-tumor gastrointestinal diseases (NTGDs). However, their causation remains unclear.

**Methods:**

We explored the causal relationship between PDs and NTGDs through bidirectional two-sample Mendelian randomization (MR) study. Large-scale genome-wide association study (GWAS) summary statistics and bidirectional two-sample MR study were used to assess the causality between PDs and NTGDs. Multiple sensitivity analyses were used to identify the robustness of our results.

**Results:**

We found that major depression was causally associated with increased risk of gastric ulcer (OR: 1.812, 95% CI: 1.320–2.487, *p* < 0.001) and irritable bowel syndrome (OR: 1.645, 95% CI: 1.291–2.097, *p* < 0.001). Meanwhile, genetically predicted gastroesophageal reflux disease contributed to the increased risk of anxiety disorders (OR: 1.425, 95% CI: 1.295–1.568, *p* < 0.001), and ulcerative colitis was related to increased risk of attention deficit/hyperactivity disorder (OR: 1.042, 95% CI: 1.008–1.078, *p* = 0.0157).

**Conclusion:**

Our study provided MR evidence to support the close causality and identify the specific direction between eight PDs and eight common NTGDs. Experimental studies to further examine the causality, underlying mechanism, and therapeutic potential of PDs and NTGDs are required.

## Introduction

Accumulating observational studies indicate the close and complicated relationship between psychiatric disorders (PDs) and non-tumor gastrointestinal diseases (NTGDs). Meanwhile, studies found that most patients with PDs are often accompanied by gastrointestinal symptoms (Kano et al., 2020; Madra et al., 2020), which demonstrates that there is a close bidirectional relationship between the central system and the digestive system, defined as “gut-brain axis.”

According to the literature, the gut and the brain interact with each other through various mechanisms, such as the nervous system, the immune system, the tryptophan metabolism as well as the gut microbiota and their metabolites. On one hand, the psychological state could affect gut homeostasis by altering the autonomic nervous system activity and modulating hormonal secretion. A disturbed psychological state could contribute to the dysregulation of the hypothalamic-pituitary-adrenal axis and the autonomic nervous system, as well as the secretion of pro-inflammatory mediators, which will furtherly facilitate the development of NTGDs (Tavakoli et al., 2021). On the other hand, the intestinal cells and microbiota can produce various bioactive substances that influence the central nervous system (Cryan et al., 2019). The compromised gut barrier and the altered gut microbiota can also result in aberrant secretion of short-chain fatty acids (SCFAs), serotonin, and translocation of gut microbiota, which exert central effects and ultimately contribute to the pathogenesis of PDs (Margolis et al., 2021). Therefore, PDs and NTGDs often co-occur, which has been corroborated by several clinical studies (Rudzki and Maes, 2020; Arp et al., 2022). For instance, depression is positively linked to an increased prevalence of intestinal inflammation and chronic diarrhea (Ballou et al., 2019; Bisgaard et al., 2022). Similarly, patients with inflammatory bowel disease (IBD) have a higher risk of anxiety and depression (Barberio et al., 2021). And some evidence indicates that bipolar disorder (BD) is associated with a higher susceptibility to irritable bowel syndrome (IBS) (Karling et al., 2016). Moreover, therapies based on this association have been widely reported. For example, the use of psychobiotics has been shown to benefit the treatment of depression and anxiety (Cheng et al., 2019), while psychological interventions have shown positive effects in the treatment of both IBD and IBS (Malhi and Mann, 2018; Camilleri, 2021).

However, due to the intricate bidirectional communication and the interplay of disease processes between these two types of diseases, it is challenging to ascertain the direction of causality between PDs and NTGDs solely based on the existing studies, which may lead to several erroneous conclusions of their causal relationship. For instance, some studies indicated that certain psychological factors, *e.g.*, depression and anxiety, elevated the risk and progression of IBD, while others have found no causal association between psychiatric disorders and ulcerative colitis (UC)/Crohn’s disease (CD) (Tavakoli et al., 2021). Meanwhile, some potential confounding factors, *e.g.*, medication use, lifestyle as well as other systemic diseases, can also impair the accuracy of the results from observational studies. Therefore, it remains many limitations for existing studies in elucidating the causal relationship between PDs and NTGDs.

Mendelian randomization (MR) is an emerging method that uses genetic variation as instrumental variables (IVs) to examine the causal effect of risk factors on disease progression. As the sequence of genetic variation and disease has been determined and conforms to the law of gene segregation, reverse causality and confounding factors can be effectively avoided, which makes the results more reliable.

We have observed that previous research has also expounded on the relationship between certain psychiatric disorders and non-tumor gastrointestinal diseases using MR methods. However, most of these studies have primarily focused on a single type of gastrointestinal disease, such as IBD (Yang et al., 2023) or IBS (Zhang et al., 2024). There are also some contradictory results, for instance, Youjie Zeng and others demonstrated that anxiety and depression do not increase the risk of gastroesophageal reflux disease (Zeng et al., 2023), while other experiments have arrived at the opposite conclusion (Chen et al., 2023; Zou et al., 2023). Therefore, in order to more comprehensively explore the potential relationship between the two, we have selected a broader range of psychiatric diseases and gastrointestinal diseases, as well as multiple datasets that differ from those used in previous studies, to provide more robust evidence.

In this study, single nucleotide polymorphisms (SNPs) of eight common PDs [attention deficit/hyperactivity disorder (ADHD), anxiety disorders, depression, major depression (MD), schizophrenia, BD, autism spectrum disorder (ASD)] and eight common NTGDs [gastric ulcer (GU), duodenal ulcer (DU), gastroduodenal ulcer (GDU), gastroesophageal reflux disease (GERD), IBS, IBD, UC, CD] were selected from the genome-wide association study (GWAS) database for a comprehensive bidirectional two-sample MR study to explore the relationship between specific PDs and NTGDs. Our study would provide a novel perspective for investigating the potential pathogenesis of the two types of diseases and the development of promising therapeutic targets.

## Materials and methods

### Study design

**FIGURE 1:**
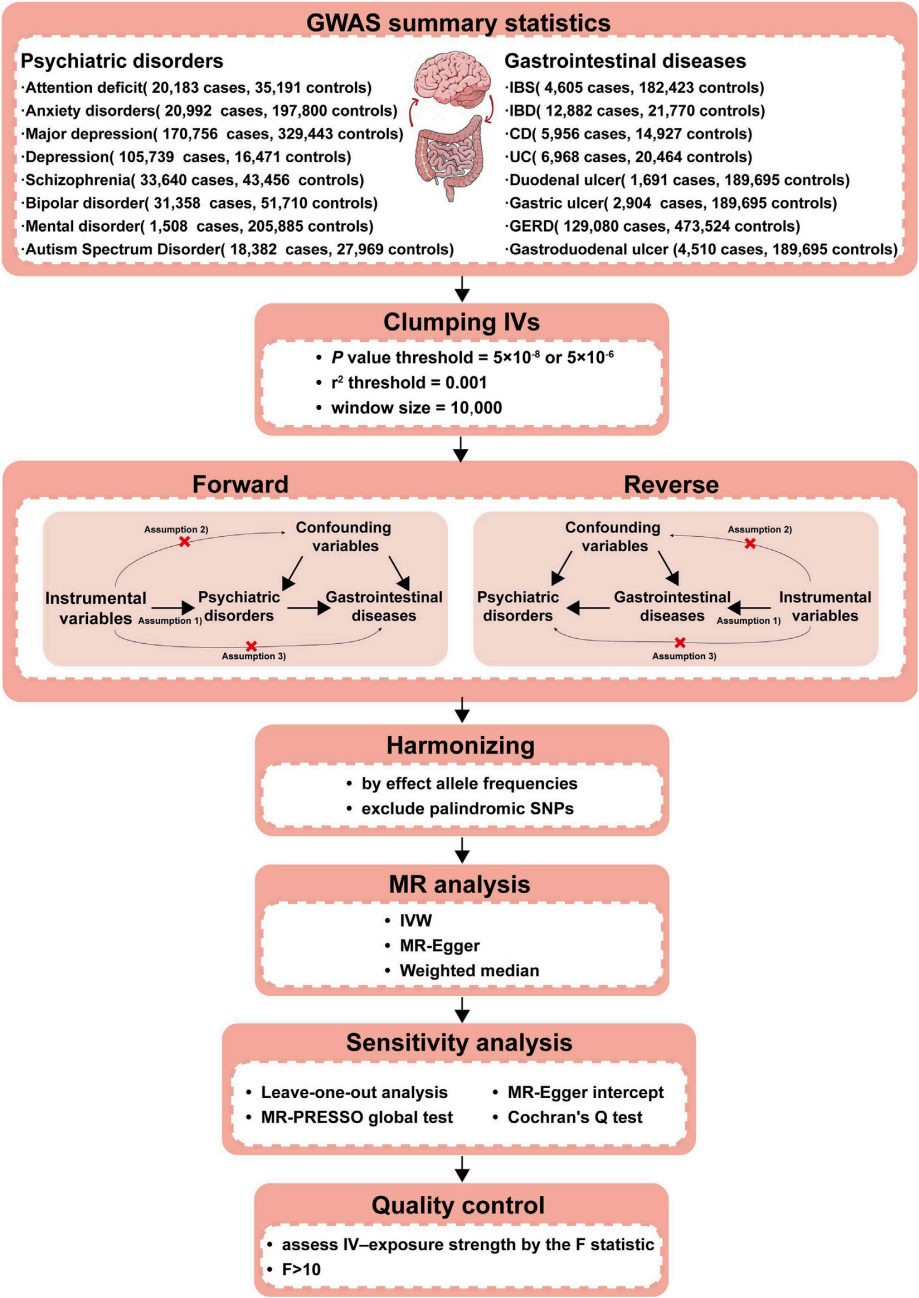
Workflow of our two-sample bidirectional MR analysis. Firstly, we searched the suitable databases and removed the data that did not meet the criteria of our research. Secondly, we further screened IVs in the database from the first step to ensure that the selected IVs met the requirements. Thirdly, to make the IVs screened in the previous step conform to the three assumptions (a. IVs are closely related to exposure; b. IVs are not associated with any confounding factors that affect the exposure-outcome association; c. IVs do not affect the outcome unless via the exposure.), we removed the IVs possibly related with confounding factors to ensure validity. Fourthly, we further excluded palindromic SNPs and harmonized the exposure and outcome datasets using effect allele frequency. Fifthly, we performed the F-statistic to ensure that no weak instruments were used. The above steps completed the identification of IVs, which were essential as the experimental basis for the subsequent research. Finally, we conducted three types of MR causal analyses and four types of sensitivity analyses using the IVs screened in the above steps to ensure that our experimental results were rigorous and reliable. CD, Crohn’s disease; IBD,inflammatory bowel disease; IBS, irritable bowel syndrome; IVs, Instrumental variables; IVW, Inverse-variance-weighted; GERD, gastroesophageal reflux disease; GWAS, genome-wide association study; MR, Mendelian randomization; MR-PRESSO, Mendelian randomization Pleiotropy RESidual Sum and Outlier; SNPs, Single nucleotide polymorphisms; UC, ulcerative colitis.

This study utilized a bidirectional two-sample MR to investigate the causal relationship between PDs and NTGDs by using genetic variants as IVs. To ensure the validity of our results, IVs should satisfy the following three assumptions: 1) strong associations with exposure factors; 2) independent from any confounding factors; 3) impact on outcome only through exposure factors. The schematic overview of the study design and data sources is shown in Figure 1.

### Data sources and study population

GWAS summary-level data for eight PDs (ADHD, anxiety disorders, depression, MD, schizophrenia, BD and ASD) were obtained from the IEU open GWAS database (https://gwas.mrcieu.ac.uk/, accessed on 10 January 2023). As for NTGDs, we selected GU, DU, GDU, GERD, IBS, and IBD (CD and UC), and assessed their GWAS data to perform MR analysis. All participants were of European ethnicity and ethical approval had been obtained in original studies. Detailed information for above GWAS data is shown in Table 1.

**TABLE 1:** The GWAS datasets of these psychiatric disorders and gastrointestinal diseases we selected.

Disease	GWAS_ID	Author	PMID	Ancestor	Cases/controls	Sample size
psychiatric disorders						
Attention deficit/hyperactivity disorder	ieu-a-1183	Demontis	NA	European	20,183/35,191	55,374
Anxiety disorders	finn-b-KRA_PSY_ANXIETY	NA	NA	European	20,992/197,800	218,792
Major depression	ieu-b-102	Howard DM	30,718,901	European	170,756/329,443	500,199
Depression	ebi-a-GCST003769	Okbay A	27,089,181	European	105,739/16,471	122,210
Schizophrenia	ieu-b-42	Ripke, S	25,056,061	European	33,640/43,456	77,096
Bipolar disorder	ieu-b-41	Stahl, E	31,043,756	European	31,358/51,710	83,068
Autism spectrum disorder	ieu-a-1185	NA	NA	European	18,382/27,969	46,351
Mental disorder	finn-b-F5_MENTALUNSPE	NA	NA	European	1,508/205,885	207,393
gastrointestinal diseases						
Duodenal ulcer	finn-b-K11_DULC	NA	NA	European	1,691/189,695	191,386
Gastric ulcer	finn-b-K11_GULC	NA	NA	European	2,904/189,695	192,599
Gastroduodenal ulcer	finn-b-K11_GASTRODUOULC	NA	NA	European	4,510/189,695	194,205
Gastroesophageal reflux disease	ebi-a-GCST90000514	Ong JS	34,187,846	European	129,080/473,524	602,604
Irritable bowel syndrome	finn-b-K11_IBS	NA	NA	European	4,605/182,423	187,028
Inflammatory bowel disease	ieu-a-31	Liu	26,192,919	European	12,882/21,770	34,652
Crohn’s disease	ieu-a-30	Liu	26,192,919	European	5,956/14,927	20,883
Ulcerative colitis	ieu-a-32	Liu	26,192,919	European	6,968/20,464	27,432

### Selection of instrumental variables

To ensure the data robustness and validity of results, the quality check was performed to screen SNPs. Firstly, the SNPs were selected from GWAS studies at thresholds for genome-wide significance level (*p* < 5 × 10^−8^), which was relaxed to 5 × 10^−6^ to avoid bias in the case that the number of sufficient SNPs was less than three. Next, *R* ^2^ > 0.001 and clumping distance <10,000 kb were set as the criteria to eliminate SNPs with linkage disequilibrium. Then, to satisfy the assumptions of IVs, SNPs associated with confounding factors were removed with the help of the phenoscanner website. Finally, we harmonized the exposure and outcome datasets using allele frequencies and removed palindrome SNPs with intermediate allele frequencies.

To avoid the instrument bias in the two-sample model, we assessed the strength of genetic instruments for exposures by F-statistic. The IVs were considered to be non-weak when F > 10. The F-statistic was calculated as F = R^2^ × [(N − 1 − k)/k] × (1 − R^2^), where R^2^ represents the degree to which a particular SNP explains the exposure, N represents the sample size, and k represents the number of SNPs used as IVs. R^2^ was calculated as R^2^ = 2 × MAF × (1−MAF) × β^2^, where MAF represents the minor allele frequency (Palmer et al., 2012).

### Statistical analysis

To elucidate the causal relationship between PDs and NTGDs, multiple methods of MR analysis and several sensitivity analyses were adopted. Inverse variance weighted (IVW) was employed as the primary method while weighted median and MR-Egger were used as auxiliary methods. Several sensitivity analyses were also performed to further test and account for horizontal pleiotropy in our MR study. Firstly, we used Cochran’s Q test to verify the heterogeneity of SNPs. Then we used the MR-Egger intercept to spot and remove the SNPs with horizontal pleiotropy. Finally, we also performed leave-one-out sensitivity test and MR-PRESSO (NbDistribution = 3,000; SignifThreshold = 0.05) to evaluate the potential pleiotropy of SNPs.

All estimates of the outcomes above were presented as odds ratio (OR) with their 95% confidence intervals (95% CI) per one standard deviation (SD) increase in the exposures. *p* < 0.05 was considered the statistical significance of evidence for potential causal effect. All the statistical analyses were performed using the “TwoSampleMR” package (version 0.5.6) and the “MRPRESSO” package (version 1.0) in R version 4.2.2.

## Results

### Instrumental variables

In the forward MR analysis, we investigated the causal relationships of PDs with NTGDs. We selected at least 9 SNPs for 4 PDs (ADHD, MD, schizophrenia, BD) as IVs at the significance level of *p* < 5 × 10^−8^ and at least 1 SNPs for the other 4 PDs (anxiety disorders, depression, ASD, mental disorder) at the significance level of *p* < 5 × 10^−6^. In the reverse MR, the causal effects of NTGDs on PDs were explored. At least 27 SNPs were identified for GERD, IBD, CD and UC as IVs (*p* < 5 × 10^−8^) while at least 5 SNPs were screened for DC, GC, GDU, and IBS as IVs (*p* < 5 × 10^−6^) (Supplementary Tables S1, S2). The F statistics of these SNPs indicated that there was almost no possibility to suffer from weak instrument bias (F > 10, Supplementary Tables S1-S3).

### The results of the main analyses and sensitivity analyses

**FIGURE 2:**
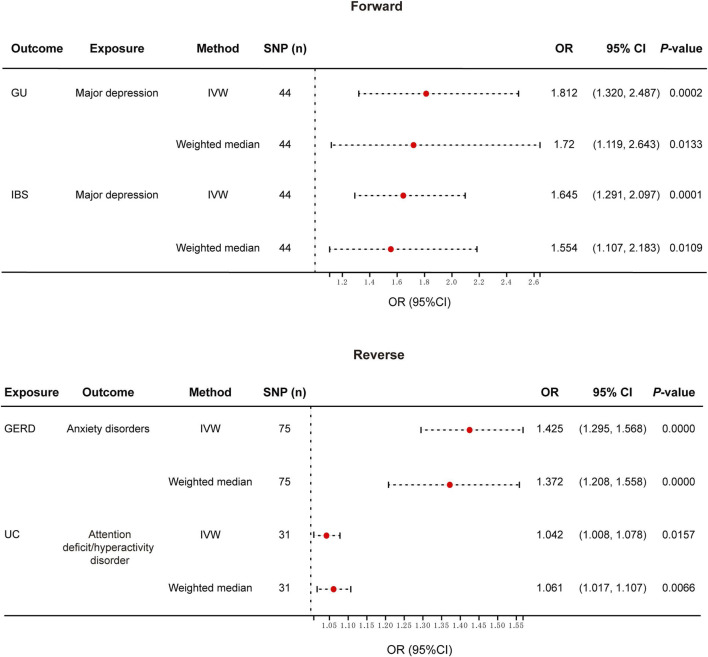
Forest plot of the causal relationship between genetically identified PDs and NTGDs in bidirectional MR analysis. IVW, Inverse-variance-weighted; SNPs, Single nucleotide polymorphisms; OR, Odds ratio; CI, confidence interval; GERD, gastroesophageal reflux disease; IBS, irritable bowel syndrome; UC, ulcerative colitis.

**FIGURE 3:**
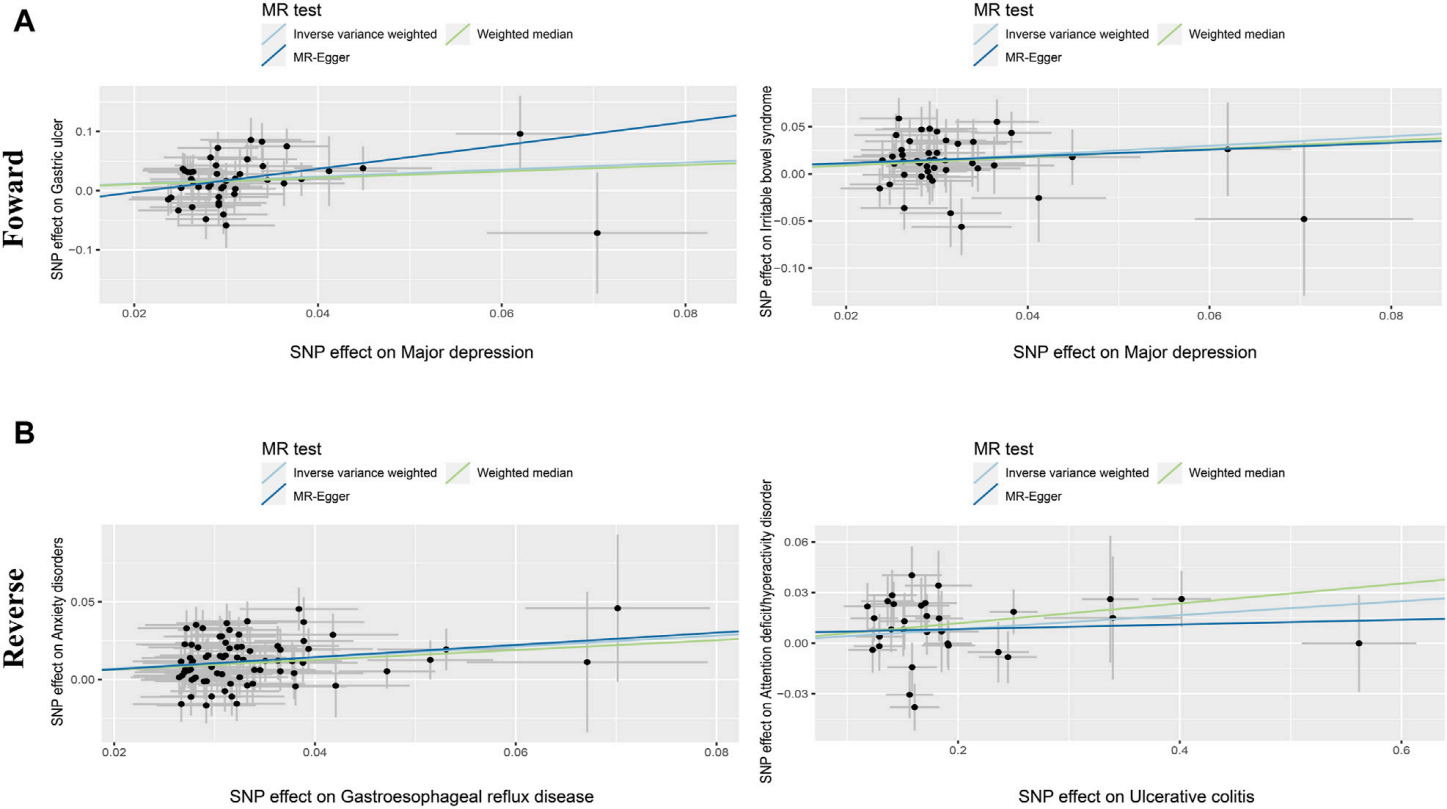
Scatter diagram of the causal relationship between genetically identified PDs and NTGDs in bidirectional MR analysis. The estimations (95%CI) were coefficients and corresponding 95% confidence interval. **(A)** Scatter plot of forward MR analysis results. **(B)** Scatter plot of reverse MR analysis results.

The overview of our MR analysis was shown in Supplementary Tables S1-S2. We identified 20 causal relationships between 8 PDs and 8 NTGDs by the IVW method. However, only 4 significant results survived after all sensitivity analyses (Figures 2, 3). Cochran’s Q statistics showed that there was no heterogeneity among the SNPs we used (*p* > 0.05). Neither MR Egger intercept detection nor MR-PRESSO detection found horizontal pleiotropy (*p* > 0.05). The removal of any SNP in the leave-one-out sensitivity test didn’t affect the results, which indicated that the positive result was not caused by a single SNP, suggesting that the results were robust (Table 2).

**TABLE 2:** All results of sensitivity analyses between genetically identified psychiatric disorders and gastrointestinal diseases.

Exposure	Outcomes	MR-PRESSO	IVW estimates		MR-Egger pleiotropy test	
		Global *p*-value	Cochran’s Q	*p*-value	MR-Egger intercept	*p*-value
Major depression	Gastric ulcer	0.353	46.325	0.337	<0.001	0.143
Major depression	Irritable bowel syndrome	0.666	39.058	0.643	0.004	0.847
Gastroesophageal reflux disease	Anxiety disorders	0.102	91.812	0.079	<0.001	0.890
Ulcerative colitis	Attention deficit/hyperactivity disorder	0.105	40.542	0.095	0.006	0.550

### The MR results for GU

After the forward MR analysis of GU, we found that MD (OR: 1.812, 95% CI: 1.320–2.487, *p* < 0.001) was associated with the risk of GU (Figures 2, 3; Supplementary Table S1) In all three causal relationship tests, MR-Egger showed results that were consistent with the direction of the results obtained by the other two methods, but not significant (OR = 7.216, *p* > 0.05). This result survived all sensitivity tests (Table 2). After the reverse MR analysis of GU, we discovered that GU increased the risk of BD (OR: 1.112, 95% CI: 1.001–1.234, *p* = 0.047). This result survived the tests of pleiotropy and heterogeneity, but failed the leave-one-out sensitivity test (Supplementary Table S2).

### The MR results for DU

In the forward MR analysis, no causality had been identified between DU and PDs (Supplementary Table S1). In the reverse MR analysis of DU, we found that DU had a protective effect on schizophrenia (OR: 0.967, 95% CI: 0.936–0.998, *p* = 0.04). Although the result survived the tests of pleiotropy and heterogeneity smoothly, it failed the leave-one-out sensitivity test (Supplementary Table S2).

### The MR results for GDU

After the forward MR analysis of GDU, we found that MD (OR: 1.365, 95% CI: 1.043–1.788, *p* = 0.0237) was associated with the risk of GDU. This result survived the test of heterogeneity but failed the test of multiplicity and leave-one-out sensitivity test (Supplementary Table S1). After the reverse MR analysis, we discovered that GDU increased the risk of depression (OR: 1.035, 95% CI: 1.007–1.064, *p* = 0.014). This result survived all the sensitivity analyses except the leave-one-out sensitivity test (Supplementary Table S2).

### The MR results for GERD

After the forward MR analysis, three PDs were identified that were causally associated with the risk of GERD, namely ADHD (OR: 1.251, 95% CI: 1.118–1.401, *p* < 0.001), depression (OR: 1.560, 95% CI: 1.114–2.184, *p* = 0.01) and MD (OR: 1.929, 95% CI: 1.741–2.138, *p* < 0.001). All the results above survived the leave-one-out sensitivity test. However, only MD survived the test of multiplicity, and none of them survived the test of heterogeneity (Supplementary Table S1). After the reverse MR analysis of GERD, we discovered that GERD increased the risk of four PD, *i.e.*, ADHD (OR: 2.112, 95% CI: 1.861–2.396, *p* < 0.001), anxiety disorders (OR: 1.425, 95% CI: 1.295–1.568, *p* < 0.001), depression (OR: 1.239, 95% CI: 1.196–1.284, *p* < 0.001) and MD (OR: 1.513, 95% CI: 1.438–1.592, *p* < 0.001). Among the three analysis methods, MR-Egger showed results that were in the same direction but not significant. All the above four results survived the leave-one-out sensitivity test (Figures 2, 3). However, depression and MD failed both heterogeneity and pleiotropy tests. ADHD survived the pleiotropy test but failed the heterogeneity test. Only anxiety disorders survived all sensitivity tests (Table 2; Supplementary Table S2).

### The MR results for IBS

After the forward MR analysis of IBS, MD (OR: 1.645, 95% CI: 1.291–2.097, *p* < 0.001) and schizophrenia (OR: 1.078, 95% CI: 1.001–1.161, *p* = 0.0463) were found to be causally associated with an increased risk of IBS (Figures 2, 3; Supplementary Table S1). The MR-Egger result of MD was consistent with the direction of the other methods, but not significant (OR = 1.432, *p* > 0.05). MD survived all the sensitivity analyses (Table 2). However, schizophrenia failed the test of multiplicity and the leave-one-out sensitivity test (Supplementary Table S1).

### The MR results for IBD and its subtypes

After the forward MR analysis of IBD, we confirmed that BD (OR: 1.158, 95% CI: 1.025–1.310, *p* = 0.0188) and schizophrenia (OR: 1.096, 95% CI: 1.029–1.169, *p* = 0.0047) increased the risk of IBD. However, in subsequent sensitivity analyses, schizophrenia failed the heterogeneity or pleiotropy test while BD failed the leave-one-out sensitivity test. Forward MR analysis of CD suggested that schizophrenia (OR: 1.100, 95% CI: 1.006–1.203, *p* = 0.0359) was associated with the increased risk of CD. But this result didn’t survive any sensitivity tests. After the forward MR analysis of UC, we found that two PDs increased the risk of UC, *i.e.,* BD (OR:1.172, 95% CI: 1.017–1.351, *p* = 0.0287) and schizophrenia (OR: 1.108, 95% CI: 1.025–1.198, *p* = 0.0097). However, in subsequent sensitivity analyses, schizophrenia didn’t pass heterogeneity or sensitivity analyses while BD didn’t survive the leave-one-out sensitivity test (Supplementary Table S1). Reverse MR analysis of UC suggested that UC increased the risk of ADHD (OR: 1.042, 95% CI: 1.008–1.078, *p* = 0.0157). The MR-Egger result was not significant, but in the same direction (OR = 1.014, *p* > 0.05). And the result survived all sensitivity tests (Figures 2, 3; Table 2).

## Discussion

In this study, we explored the potential causal associations between eight PDs and eight common NTGDs at the genetic level using MR analyses based on large-scale GWAS summary statistics. On the ground of the forward MR analysis, we discovered that the genetic susceptibility to MD is related to an elevated risk of GU and IBS. And in reverse MR studies, we demonstrated that genetic susceptibility to GERD increased the risk of anxiety. Meanwhile, UC, a subtype of IBD, was found to increase the risk of ADHD. Although the MR-Egger results were not significant, MR-Egger is a conservative method when used as a causal analysis method, and its accuracy and stability are low. When the MR-Egger result has no statistical significance but is consistent with the IVW result, it does not provide additional evidence for the causal effect, but it also does not contradict or reduce the credibility of the original result (Burgess and Thompson, 2017).

Mendelian randomization, as a reliable research method, provides evidence that is second only to randomized controlled trials in terms of reliability, yet it is more expedient, cost-effective, and effectively addresses the ethical barriers encountered in randomized controlled trials. It holds significant value for causal research across various diseases. Based on the principles of Mendelian randomization, we utilized genetic variants associated with the exposure as instrumental variables. The random allocation of genetic alleles mimics the random assignment of groups in a randomized controlled trial, thus ensuring the approximate equal distribution of potential confounding factors between groups. If the genetic variation associated with the exposure correlates with differences in outcomes, a causal relationship between exposure and outcome can be inferred. Moreover, since genetic variations are inherent, the sequence of exposure and outcome is predetermined, effectively mitigating the interference of reverse causation and enhancing the reliability and stability of the study results.

IBS is a kind of functional disorders characterized by the altered gastrointestinal movement and often results in abnormal bowel patterns without any organic lesions (Camilleri, 2021). Epidemiological studies have shown that individuals with MD have a higher incidence of IBS (Ballou et al., 2019). MD is a prevalent psychiatric disorder characterized by a low mood and accompanied by various physical symptoms (Malhi and Mann, 2018). Several studies indicated that MD could affect the development and progression of IBS through multiple mechanisms. Firstly, patients during MD episode showed increased microglial activation which is identified to promote colorectal distension and significantly induce visceral hypersensitivity by animal experiments (Bradesi et al., 2009; Zhang et al., 2016). Furthermore, visceral hypersensitivity might contribute to IBS pathogenesis (Kuiken et al., 2005; Elsenbruch et al., 2010). Besides, MD often contributed to the abnormal activation of several inflammatory factors (Gadad et al., 2018; Beurel et al., 2020) as well as the dysregulation of gut immunity, which further disturbed gut homeostasis and altered the intestinal movement (Ng et al., 2018). Despite the lack of definitive evidence for the precise immune response pattern, numerous studies have demonstrated a strong correlation between IBS and intestinal inflammation (Ng et al., 2018). Furthermore, patients suffering from MD frequently display autonomic dysregulation (Herbsleb et al., 2019), which might further enhance gut sensitivity, stimulate mast cells, lead to cytokine imbalance, and impair intestinal barrier (Stasi et al., 2012). In this MR study, we also demonstrated that MD elevated the likelihood of IBS at the genetic level. Nevertheless, the specific molecular mechanisms remain to be verified by further experiments.

Additionally, our study revealed a causal connection between MD and an elevated risk of GU. GU, a disorder resulting from inflammatory impairments in the gastric mucosa, is intimately associated with various stress factors (Sverdén et al., 2019). An increasing number of researches demonstrated that MD might facilitate GU progression through various mechanisms, *e.g.*, stimulating secretion of gastric acid, impairing the protection of gastric mucosa, enhancing the susceptibility to *H. pylori* infection, and so forth (Overmier and Murison, 2013). These prior findings provided a robust empirical basis for the validity of the conclusions in our study.

Meanwhile, our research indicated that NTGDs may also have a reciprocal impact on the emergence of some PDs. Firstly, intestinal mucosal cells and gut microbiota could produce metabolites that can directly influence the nervous system or cross the blood-brain barrier, thus affecting the brain. Therefore, disturbed intestinal homeostasis can abnormally activate the brain-gut axis and facilitate PDs development (Cryan et al., 2019). For instance, our MR research revealed that patients with GERD showed a higher risk of anxiety disorders. GERD is a disease caused by the reflux of stomach contents with a variety of concurrent symptoms (Fass, 2022). Patients with GERD always exhibited elevated levels of pro-inflammatory factors in the esophageal mucosa, which might indirectly induce central neuroinflammation and therefore contribute to the pathogenesis of anxiety disorders (He et al., 2022).

Moreover, GERD patients are often accompanied by the gut microbiota alteration, such as the increased abundance of *Lactobacillus*, which was reported to be positively correlated with anxiety (Simpson et al., 2021). These prior studies support the hypothesis of increased susceptibility to anxiety disorders in patients with GERD. Concomitantly, several observational studies have corroborated our findings (Mohammad et al., 2019; Bai et al., 2021; He et al., 2022). This result highlights the importance of timely treatment in patients with GERD in order to avoid anxiety disorders. Meanwhile, we also found that patients with UC had a higher risk of ADHD. As a subtype of IBD confined to the colonic mucosa and submucosa (Sun et al., 2021), UC patients also existed significant gut microbiota dysbiosis. It might facilitate the onset of ADHD by modulating catecholamine responsiveness and aggravating oxidative stress (Bull-Larsen and Mohajeri, 2019; Boonchooduang et al., 2020; Checa-Ros et al., 2021). However, there remain inconsistent results in previous clinical studies and the sample sizes are obviously limited. The underlying specific mechanisms warrant further investigation.

With the growing interest in the brain-gut axis recently, the link between PDs and NTGDs has been uncovered by an increasing number of studies. However, the presence of confounding factors and bidirectional causality bring challenges to elucidate the true association between the two. In this study, we first utilized large-scale MR analysis to verify the causal relationship and the direction of causality between specific PDs and NTGDs, and evaluated the robustness by conducting multiple sensitivity analyses (*e.g.,* Cochran’s Q test, MR-Egger pleiotropy test, leave-one-out sensitivity test, and MR-PRESSO detection). Our finding would assist in comprehending the intricate interplay between these two types of diseases, and thus provide new insight for investigating the potential pathogenesis and therapeutic targets of PDs and NTGDs.

However, our study still suffers from several limitations. Firstly, due to the lack of relevant statistical data in the disease database we utilized, we were unable to analyze the relationship between mental disorders and NTGDs at different stages. Secondly, we only examined the causal relationships of common psychiatric disorders, potentially overlooking certain types of mental illnesses that might have causal links to digestive disorders. Thirdly, despite efforts to eliminate known common confounders in this experiment, the presence of unknown confounding factors cannot be entirely ruled out, thereby impacting the reliability of the results. Lastly, all the data used in this study are derived from European populations, which might impose certain constraints when extrapolating the results to other ethnicities.

## Conclusion

To sum up, we uncovered the causal relationship among eight PDs (ADHD, anxiety, depression, MD, schizophrenia, BD, and ASD) and eight NTGDs (GU, DU, GDU, GERD, IBS, IBD, CD, and UC). Our discoveries offer insights into the intricate interplay between two types of diseases and the investigation of the potential pathogenesis and therapeutic targets. However, the specific mechanisms between PDs and NTGDs remain to be further elucidated in the future.

## Significant outcomes

• There is the close relationship between psychiatric disorders (PDs) and non-tumor gastrointestinal diseases (NTGDs).
• The genetic susceptibility to major depression is significantly related to the elevated risk of gastric ulcer and irritable bowel syndrome.
• The genetic susceptibility to gastroesophageal reflux disease contributed to the increased risk of anxiety disorders.

## Limitations

• Interaction between PDs and NTGDs at different disease stages failed to be analyzed due to the lack of incomplete information in the databases.
• We only examined the causal relationships of common psychiatric disorders, potentially overlooking certain types of mental illnesses that might have causal links to digestive disorders.
• Despite efforts to eliminate known common confounders in this experiment, the presence of unknown confounding factors cannot be entirely ruled out, thereby impacting the reliability of the results.
• The data used in our experiments was from European populations to avoid population stratification bias but might lead to some constraints to generalizing our findings to other ethnic groups.

## Data Availability

The original contributions presented in the study are included in the article/Supplementary Material, further inquiries can be directed to the corresponding authors.

## Supplementary material

SUPPLEMENTARY FIGURE S1 Bidirectional MR leave-one-out analysis.

SUPPLEMENTARY TABLE S1 Causal effects of PDs on NTGDs.

SUPPLEMENTARY TABLE S2 Causal effects of NTGDs on PDs.

SUPPLEMENTARY TABLE S3 The instrumental variables of exposures which had significant causal effects in bidirectional MR analyses and survived after sensitivity analyses.

## References

[B1] Arp L. Jansson S. Wewer V. Burisch J. (2022). Psychiatric disorders in adult and paediatric patients with inflammatory bowel diseases - a systematic review and meta-analysis. J. Crohn's Colitis 16 (12), 1933–1945. 10.1093/ecco-jcc/jjac095 35775920

[B2] Bai P. Bano S. Kumar S. Sachdev P. Ali A. Dembra P. (2021). Gastroesophageal reflux disease in the young population and its correlation with anxiety and depression. Cureus 13 (5), e15289. 10.7759/cureus.15289 34194886 PMC8236209

[B3] Ballou S. Katon J. Singh P. Rangan V. Lee H. N. McMahon C. (2019). Chronic diarrhea and constipation are more common in depressed individuals. Clin. Gastroenterology Hepatology 17 (13), 2696–2703. 10.1016/j.cgh.2019.03.046 PMC6776710 30954714

[B4] Barberio B. Zamani M. Black C. J. Savarino E. V. Ford A. C. (2021). Prevalence of symptoms of anxiety and depression in patients with inflammatory bowel disease: a systematic review and meta-analysis. Lancet. Gastroenterology Hepatology 6 (5), 359–370. 10.1016/S2468-1253(21)00014-5 33721557

[B5] Beurel E. Toups M. Nemeroff C. B. (2020). The bidirectional relationship of depression and inflammation: double trouble. Neuron 107 (2), 234–256. 10.1016/j.neuron.2020.06.002 32553197 PMC7381373

[B6] Bisgaard T. H. Allin K. H. Keefer L. Ananthakrishnan A. N. Jess T. (2022). Depression and anxiety in inflammatory bowel disease: epidemiology, mechanisms and treatment. Nat. Rev. Gastroenterology Hepatology 19 (11), 717–726. 10.1038/s41575-022-00634-6 35732730

[B7] Boonchooduang N. Louthrenoo O. Chattipakorn N. Chattipakorn S. C. (2020). Possible links between gut-microbiota and attention-deficit/hyperactivity disorders in children and adolescents. Eur. J. Nutr. 59 (8), 3391–3403. 10.1007/s00394-020-02383-1 32918136

[B8] Bradesi S. Svensson C. I. Steinauer J. Pothoulakis C. Yaksh T. L. Mayer E. A. (2009). Role of spinal microglia in visceral hyperalgesia and NK1R up-regulation in a rat model of chronic stress. Gastroenterology 136 (4), 1339–1348. 10.1053/j.gastro.2008.12.044 19249394 PMC2812027

[B9] Bull-Larsen S. Mohajeri M. H. (2019). The potential influence of the bacterial microbiome on the development and progression of ADHD. Nutrients 11 (11), 2805. 10.3390/nu11112805 31744191 PMC6893446

[B10] Burgess S. Thompson S. G. (2017). Interpreting findings from Mendelian randomization using the MR-Egger method. Eur. J. Epidemiol. 32 (5), 377–389. 10.1007/s10654-017-0255-x 28527048 PMC5506233

[B11] Camilleri M. (2021). Diagnosis and treatment of irritable bowel syndrome: a review. JAMA 325 (9), 865–877. 10.1001/jama.2020.22532 33651094

[B12] Checa-Ros A. Jeréz-Calero A. Molina-Carballo A. Campoy C. Muñoz-Hoyos A. (2021). Current evidence on the role of the gut microbiome in ADHD pathophysiology and therapeutic implications. Nutrients 13 (1), 249. 10.3390/nu13010249 33467150 PMC7830868

[B13] Chen D. Zhang Y. Huang T. Jia J. (2023). Depression and risk of gastrointestinal disorders: a comprehensive two-sample Mendelian randomization study of European ancestry. Psychol. Med. 53 (15), 7309–7321. 10.1017/S0033291723000867 37183395

[B14] Cheng L.-H. Liu Y.-W. Wu C.-C. Wang S. Tsai Y.-C. (2019). Psychobiotics in mental health, neurodegenerative and neurodevelopmental disorders. J. Food Drug Analysis 27 (3), 632–648. 10.1016/j.jfda.2019.01.002 PMC9307042 31324280

[B15] Cryan J. F. O'Riordan K. J. Cowan C. S. M. Sandhu K. V. Bastiaanssen T. F. S. Boehme M. (2019). The microbiota-gut-brain Axis. Physiol. Rev. 99 (4), 1877–2013. 10.1152/physrev.00018.2018 31460832

[B16] Elsenbruch S. Rosenberger C. Enck P. Forsting M. Schedlowski M. Gizewski E. R. (2010). Affective disturbances modulate the neural processing of visceral pain stimuli in irritable bowel syndrome: an fMRI study. Gut 59 (4), 489–495. 10.1136/gut.2008.175000 19651629

[B17] Fass R. (2022). Gastroesophageal reflux disease. N. Engl. J. Med. 387 (13), 1207–1216. 10.1056/NEJMcp2114026 36170502

[B18] Gadad B. S. Jha M. K. Czysz A. Furman J. L. Mayes T. L. Emslie M. P. (2018). Peripheral biomarkers of major depression and antidepressant treatment response: current knowledge and future outlooks. J. Affect. Disord. 233, 3–14. 10.1016/j.jad.2017.07.001 28709695 PMC5815949

[B19] He M. Wang Q. Yao D. Li J. Bai G. (2022). Association between psychosocial disorders and gastroesophageal reflux disease: a systematic review and meta-analysis. J. Neurogastroenterol. Motil. 28 (2), 212–221. 10.5056/jnm21044 35362447 PMC8978133

[B20] Herbsleb M. Schumann A. Lehmann L. Gabriel H. H. W. Bär K.-J. (2019). Cardio-respiratory fitness and autonomic function in patients with major depressive disorder. Front. Psychiatry 10, 980. 10.3389/fpsyt.2019.00980 32116813 PMC7011194

[B21] Kano M. Muratsubaki T. Yagihashi M. Morishita J. Mugikura S. Dupont P. (2020). Insula activity to visceral stimulation and endocrine stress responses as associated with alexithymia in patients with irritable bowel syndrome. Psychosom. Med. 82 (1), 29–38. 10.1097/PSY.0000000000000729 31609924

[B22] Karling P. Maripuu M. Wikgren M. Adolfsson R. Norrback K.-F. (2016). Association between gastrointestinal symptoms and affectivity in patients with bipolar disorder. World J. Gastroenterology 22 (38), 8540–8548. 10.3748/wjg.v22.i38.8540 PMC5064035 27784966

[B23] Kuiken S. D. Lindeboom R. Tytgat G. N. Boeckxstaens G. E. (2005). Relationship between symptoms and hypersensitivity to rectal distension in patients with irritable bowel syndrome. Alimentary Pharmacol. Ther. 22 (2), 157–164. 10.1111/j.1365-2036.2005.02524.x 16011674

[B24] Madra M. Ringel R. Margolis K. G. (2020). Gastrointestinal issues and autism spectrum disorder. Child Adolesc. Psychiatric Clin. N. Am. 29 (3), 501–513. 10.1016/j.chc.2020.02.005 PMC8608248 32471598

[B25] Malhi G. S. Mann J. J. (2018). Depression. Lancet 392 (10161), 2299–2312. 10.1016/S0140-6736(18)31948-2 30396512

[B26] Margolis K. G. Cryan J. F. Mayer E. A. (2021). The microbiota-gut-brain Axis: from motility to mood. Gastroenterology 160 (5), 1486–1501. 10.1053/j.gastro.2020.10.066 33493503 PMC8634751

[B27] Mohammad S. Chandio B. Soomro A. A. Lakho S. Ali Z. Ali Soomro Z. (2019). Depression and anxiety in patients with gastroesophageal reflux disorder with and without chest pain. Cureus 11 (11), e6103. 10.7759/cureus.6103 31763106 PMC6858267

[B28] Ng Q. X. Soh A. Y. S. Loke W. Lim D. Y. Yeo W.-S. (2018). The role of inflammation in irritable bowel syndrome (IBS). J. Inflamm. Res. 11, 345–349. 10.2147/JIR.S174982 30288077 PMC6159811

[B29] Overmier J. B. Murison R. (2013). Restoring psychology's role in peptic ulcer. Appl. Psychol. Health Well-being 5 (1), 5–27. 10.1111/j.1758-0854.2012.01076.x 23457084 PMC3613748

[B30] Palmer T. M. Lawlor D. A. Harbord R. M. Sheehan N. A. Tobias J. H. Timpson N. J. (2012). Using multiple genetic variants as instrumental variables for modifiable risk factors. Stat. Methods Med. Res. 21 (3), 223–242. 10.1177/0962280210394459 21216802 PMC3917707

[B31] Rudzki L. Maes M. (2020). The microbiota-gut-immune-glia (MGIG) Axis in major depression. Mol. Neurobiol. 57 (10), 4269–4295. 10.1007/s12035-020-01961-y 32700250

[B32] Simpson C. A. Diaz-Arteche C. Eliby D. Schwartz O. S. Simmons J. G. Cowan C. S. M. (2021). The gut microbiota in anxiety and depression - a systematic review. Clin. Psychol. Rev. 83, 101943. 10.1016/j.cpr.2020.101943 33271426

[B33] Stasi C. Rosselli M. Bellini M. Laffi G. Milani S. (2012). Altered neuro-endocrine-immune pathways in the irritable bowel syndrome: the top-down and the bottom-up model. J. Gastroenterology 47 (11), 1177–1185. 10.1007/s00535-012-0627-7 22766747

[B34] Sun Y. Zhang Z. Zheng C.-Q. Sang L.-X. (2021). Mucosal lesions of the upper gastrointestinal tract in patients with ulcerative colitis: a review. World J. Gastroenterology 27 (22), 2963–2978. 10.3748/wjg.v27.i22.2963 PMC8192286 34168401

[B35] Sverdén E. Agréus L. Dunn J. M. Lagergren J. (2019). Peptic ulcer disease. BMJ 367, l5495. 10.1136/bmj.l5495 31578179

[B36] Tavakoli P. Vollmer-Conna U. Hadzi-Pavlovic D. Grimm M. C. (2021). A review of inflammatory bowel disease: a model of microbial, immune and neuropsychological integration. Public Health Rev. 42, 1603990. 10.3389/phrs.2021.1603990 34692176 PMC8386758

[B37] Yang X. Yang L. Zhang T. Zhang H. Chen H. Zuo X. (2023). Causal atlas between inflammatory bowel disease and mental disorders: a bi-directional 2-sample Mendelian randomization study. Front. Immunol. 14, 1267834. 10.3389/fimmu.2023.1267834 37901213 PMC10611497

[B38] Zeng Y. Cao S. Yang H. (2023). The causal role of gastroesophageal reflux disease in anxiety disorders and depression: a bidirectional Mendelian randomization study. Front. Psychiatry 14, 1135923. 10.3389/fpsyt.2023.1135923 36911112 PMC9992201

[B39] Zhang G. Zhao B.-X. Hua R. Kang J. Shao B.-M. Carbonaro T. M. (2016). Hippocampal microglial activation and glucocorticoid receptor down-regulation precipitate visceral hypersensitivity induced by colorectal distension in rats. Neuropharmacology 102, 295–303. 10.1016/j.neuropharm.2015.11.028 26656865

[B40] Zhang T. Chen Y. Li X. Zhang J. Duan L. (2024). Genetic associations and potential mediators between psychiatric disorders and irritable bowel syndrome: a Mendelian randomization study with mediation analysis. Front. Psychiatry 15, 1279266. 10.3389/fpsyt.2024.1279266 38352653 PMC10861787

[B41] Zou M. Zhang W. Shen L. Xu Y. Zhu Y. (2023). Major depressive disorder plays a vital role in the pathway from gastroesophageal reflux disease to chronic obstructive pulmonary disease: a Mendelian randomization study. Front. Genet. 14, 1198476. 10.3389/fgene.2023.1198476 37404328 PMC10315650

