# Supplementary figures and images for: A comprehensive Mendelian randomization study highlights the relationship between psychiatric disorders and non-tumor gastrointestinal diseases
Source: Front Genet. 2024 May 9;15:1392518. doi: 10.3389/fgene.2024.1392518 (PMC11129081; doi:10.3389/fgene.2024.1392518)

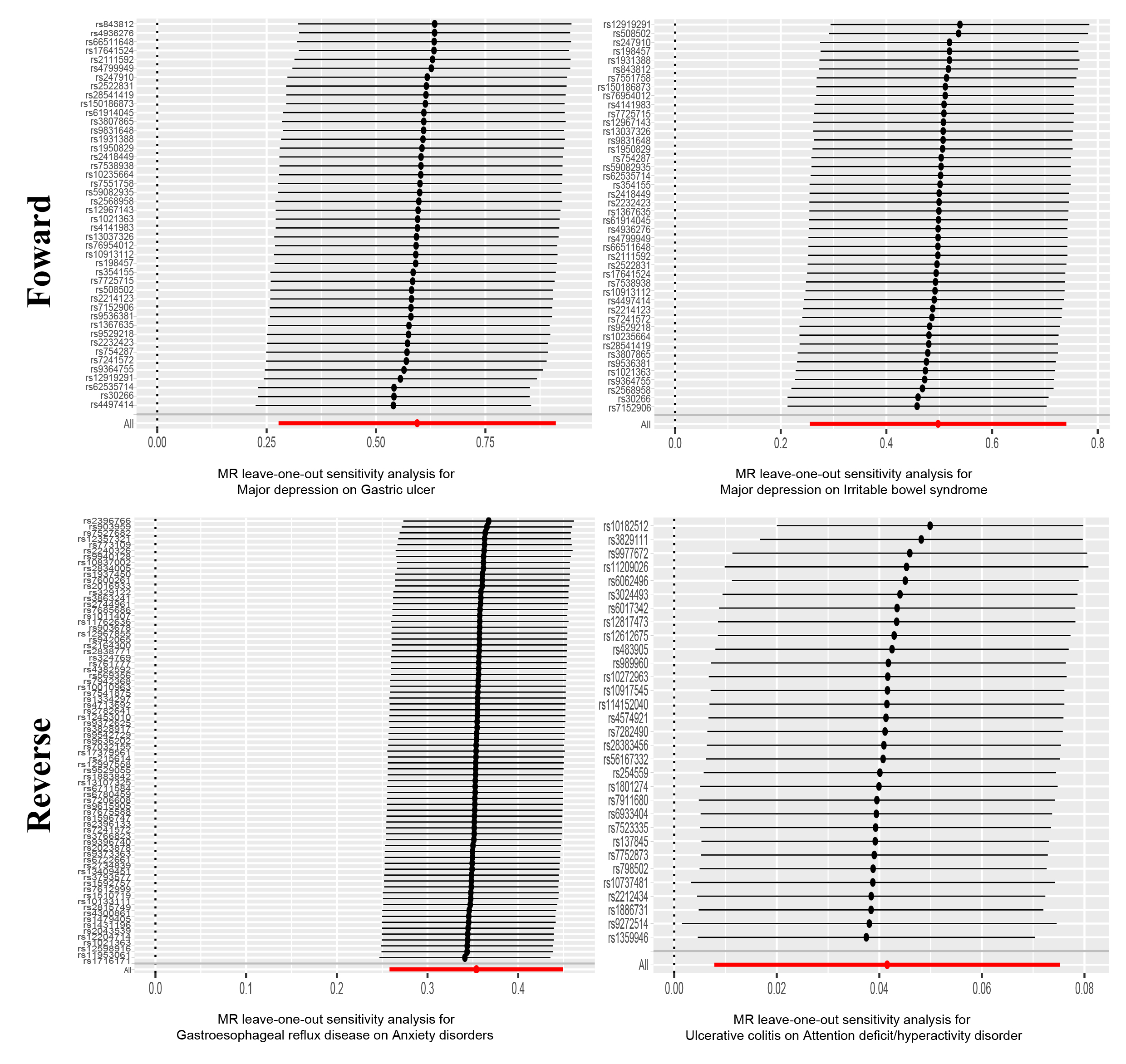

Supplement: Supplementary file 1 [file Image1.JPEG]
